# Sunitinib and Sorafenib Modulating Antitumor Immunity in Hepatocellular Cancer

**Published:** 2018-06-08

**Authors:** Dai Liu, Xiaoqiang Qi, Yariswamy Manjunath, Eric T. Kimchi, Lixin Ma, Jussuf T. Kaifi, Kevin F. Staveley-O’Carroll, Guangfu Li

**Affiliations:** 1 Department of Surgery, University of Missouri-Columbia, Columbia; 2 Ellis Fischel Cancer Center, University of Missouri-Columbia, Columbia; 3 Department of Radiology, University of Missouri-Columbia, Coumbia, MO65212, Harry S. Truman Memorial VA Hospital Biomolecular Imaging Center, USA; 4 Department of Molecular Microbiology and Immunology, University of Missouri-Columbia, Columbia

**Keywords:** Hepatocellular cancer (HCC), Sunitinib, Sorafenib, Chemoimmunotherapy, Regulatory T cells (Tregs), Myeloid-derived suppressive cells (MDSCs)

## Abstract

Sorafenib and sunitinib are multiple tyrosine kinase inhibitors. Both of them have been approved by the US FDA in the treatment of patients with malignancies. In order to develop an effective and clinically useful chemoimmunotherapy modality against hepatocellular cancer (HCC), we investigate their tumoricidal and immune modulatory effect in the setting of HCC. *In vitro* experiments suggested that sunitinib and sorafenib both induced HCC cell apoptosis at an equivalent level, but stronger suppressive function to cell proliferation was detected in sorafenib. Correspondingly, treatment of tumor-bearing mice with sorafenib led to the suppression of tumor growth to a larger extent than sunitinib. Flow cytometry showed that treatment with sunitinib, not sorafenib, significantly reduced the frequency of regulatory T cells (Tregs) and myeloid-derived suppressive cells (MDSCs) in tumor-bearing mice; and allowed splenic lymphocytes to produce equivalent levels of IFN-γ and TNF-α in response to vaccination as that in wild type mice. This activation was not detected in control and sorafenib-treated tumor mice. In addition, treatment of tumor-bearing mice with sunitinib followed by adoptive transfer of tumor antigen-specific CD8^+^ T cells and immunization resulted in the additional suppression to tumor growth compared to sunitinib monotherapy. These results imply treatment with sunitinib, not sorafenib, is able to prevent tumor-induced immunotolerance and activate antitumorimmunity. Our data suggest that sunitinib may be a preferable chemotherapeutic agent to use in combination with immunotherapy for the treatment of HCC.

## INTRODUCTION

Hepatocellular cancer (HCC) is a second leading cause of cancer death worldwide [1]. The incidence and mortality of HCC continue to increase in the United States (US) [2]. The currently available therapeutic options only provide limited benefit [3,4]. In the last few decades immunotherapy has become an important part of treating cancer [5]. Targeting Cytotoxic T-lymphocyte-associated protein 4 (CTLA-4), programmed death-1 (PD-1), and programmed death-1 ligand (PD-L1) has generated successful immunotherapeutic interventions [6–8]. Antibodies against PD-1, CTLA-4, and PD-L1 were recently approved by the US FDA in the treatment of patients with advanced melanoma [9] and squamous non-small cell lung cancer et al. [10]. This clinical breakthrough encourages the translation of immunotherapies to other cancers including HCC [3,11].

However, up to date, only few clinical trials have been performed in patients with HCC and clinical outcome is disappointing [12]. An intrinsic immune suppressive microenvironment represents a major impediment [4]. One promising immune-based therapeutic modality of HCC is chemoimmunotherapy [13] in which chemotherapy not only exerts inherent tumoricidal effect but also restores the ability of immune system to destroy the established tumors [13,14]. In the present study, we compare the role of FDA-approved chemotherapeutic drugs sunitinib [13,14] and sorafenib [15] in overcoming tumor-induced immunotolerance and synergizing with immunotherapy in the treatment of HCC.

Sorafenib (Bayer Pharmaceuticals, West Haven, CT) and sunitinib (Pfizer Inc., New York, NY) are small molecular inhibitors of multiple tyrosine kinases. Both of them displaying similar drug profiles and overlapping targets, have been approved by the US FDA for advanced renal cell cancer (RCC) [16,17]. In 2008, sorafenib became the first and only systemically administered therapy for unresectable HCC, as it increases the median overall survival of patients from 7.9 to 10.7 months [18]. In 2013, one group conducted an open-label, phase III trial to compare the therapeutic effect of sunitinib and sorafenibin HCC. The results indicated the overall survival with sunitinib was not superior or equivalent to sorafenib [19]. With the development of immunotherapy over the past several years, evaluating the effect of sunitinib and sorafenib in antitumor immunity in the context of HCC towards development of curative chemoimmunotherapy has gained increasing interest. Using new clinically relevant murine model established recently by us, we assess the role of sunitinib and sorafenib in antitumor immunity in the setting of HCC and investigate each monotherapy and the combination with adoptive transfer of tumor antigen-specific CD8^+^ T cells in the treatment of HCC.

## MATERIALS AND METHODS

### Cell line, cell proliferation, and apoptosis assay

Human hepatocellular carcinoma cell line HepG2 and human hepatoma cell line SkHep1 were obtained from American Type Culture Collection (Manassas, VA), and grown in MEM with 10% FBS at 37°C in 5% CO_2_ humidified atmosphere. B6/WT-19 cell is a transformed C57BL/6 mouse embryofibroblast line that expresses wild-type SV40 T antigen (TAg). 2 ×10^4^ Sk-Hep1 or HepG2 cells were seeded into each well of 96-well plate, then treated with the indicated concentrations of sunitinib or sorafenib. Cell proliferation and apoptosis assays at the indicated time were conducted with the Proliferation Assay Kit (Promega) and Apo-one Homogeneous Caspase-3/7 Assay kit (Promega) according to the manufacturer’s instructions.

### Mice

Line MTD2 [20] and 416 [21] mice served as the source of tumorigenic hepatocytes and tumor antigen-specific (TAS) CD8^+^ T cells (TCR-I T cells), respectively. Male C57BL/6 mice from Jackson Laboratory (Bar Harbor, ME) were used as recipient mice in our HCC model. Animal experiments were approved by the ACUC of University of Missouri-Columbia. All mice received humane care according to the criteria outlined in the “Guide for the Care and Use of Laboratory Animals”.

### IP administration of CCl_4_, ISPL injection of hepatocytes, and magnetic resonance imaging (MRI)

10% CCl_4_ (v/v) solution in corn oil was intraperitoneally (IP) injected into C57BL/6 mice twice a week at 8 mL/kg of body weight (BW) for six weeks. Two weeks after last injection, the mice received TAg-transgenic hepatocytes isolated from young male MTD2 mice by intrasplenic (ISPL) injection [13]. Briefly, C57BL/6 mice under general anesthesia underwent a ½ cm flank incision. Two 10 mm titanium clips were placed between the upper and lower branch of the splenic vasculature and spleen was cut between the two clips. Hepatocytes were injected into the lower pole of the spleen. The lower pole of the spleen was removed following injection. All MRI scans for tumor surveillance were obtained on a 7.0 Tesla system (Bruker Biospin, Billerica, MA, USA) with in-plan resolution 0.1 mm and slice thickness 1 mm.

### *In vivo* treatment of tumor-bearing mice with sunitinib or sorafenib and immunization with B6/WT-19 cells

Sunitinib was orally administrated to each mouse at 40 mg/kg of BW in 0.2 mL every other day for two weeks. Sorafenib was orally administrated to each mouse at 30 mg/ml daily for 2 weeks. For immunization, 3 × 10^7^ B6/WT-19 cells freshly harvested were suspended in 0.2 mL of PBS and IP injected into each mouse [13].

### Isolation and purification of TCR-I transgenic T cells and the adoptive transfer

416 mouse is a transgenic strain carrying a rearranged TCR transgene specific for the H2-Db-restricted TAg epitope I (residues 206-215: SAINNYAQKL). These mice are now available from the Jackson Laboratory as line B6.Cg-Tg (TcraY1,TcrbY1) 416Tev/J. Transgene positive TCR-I progenies were identified by staining peripheral blood lymphocytes with FITC-labeled anti-Vβ7 antibody (BD Pharmingen). In the present studies, 12-week old 416 mice were euthanized to isolate spleen or lymph nodes for isolating lymphocytes. CD8^+^ TCR-I T cells were enriched by MACS sorting using CD8^+^ magnetic microbeads (Miltenyi Biotech, Auburn, CA) according to the manufacturer’s instructions. CD8-enriched cells were stained with anti-CD8 and Db/I tetramer to determine purity, which ranged between 85–90%. 1 × 10^6^ purified TCR-I T cells were suspended in 0.2 mL of HBSS and injected into the mice via tail vein.

### Flow cytometric analysis

*Ex vivo* staining of splenic lymphocytes with fluorochrome-labeled antibodies was performed on single-cell suspensions [14]. Stained cells were analyzed with a FACScan flow cytometer (BD Biosciences). Data were analyzed using FlowJo software (Tree Star). Staining for intracellular IFN-γ and TNF-α was performed as described previously [13]. Staining for FoxP3 was performed with a buffer set from eBioscience.

### Statistics

Paired data were analyzed using a 2-tailed paired Student’s *t* test. A *p* value of less than 0.05 was considered significant.

## RESULTS

### Sunitinib and sorafenib suppress HCC and hepatoma cell growth *in vitro*

**Figure 1:**
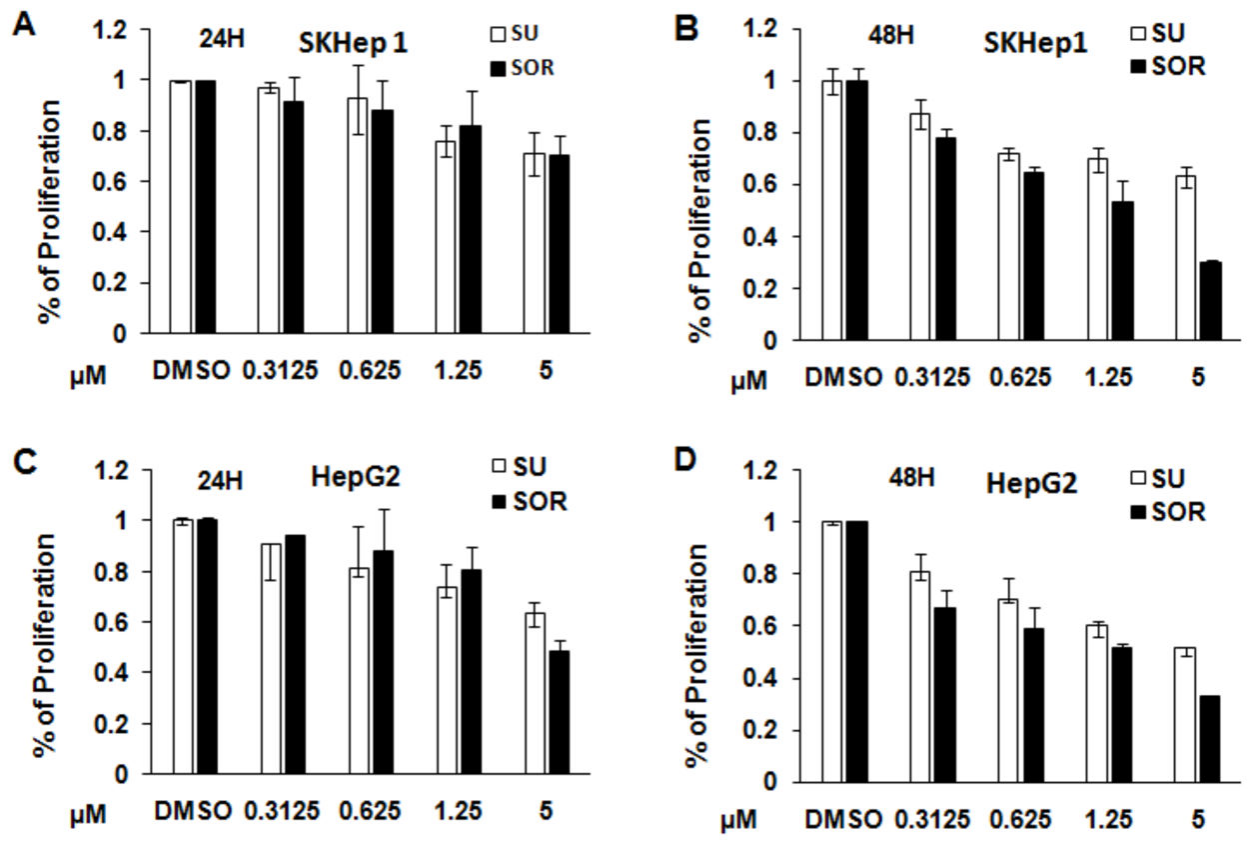
Sunitinib and sorafenib inhibit the proliferation of human HepG2 and Sk-Hep 1 cells in a time- and dose-dependent manner HepG2, a human HCC cell, and Sk Hep 1, a human hepatic adenocarcinoma cell, were seeded into 96-well plates at 2 ×10^4^ cells per well in completed medium. The second day, sunitinib or sorafenib was added into each well at the indicated concentrations, DMSO as a vehicle solution was used as control. 24 or 48 hours after the treatment, cell proliferation of Sk-Hep1 (A, B) and HepG2 (C, D) were measured with Cell’-Titer 96 Aqueous One Solution Cell Proliferation Assay Kit (Promega) according to the manufacturer’s instructions. The results are presented as a percentage of control cells treated with vehicle DMSO. n=3; error bars represent means ± S.D.

**Figure 2:**
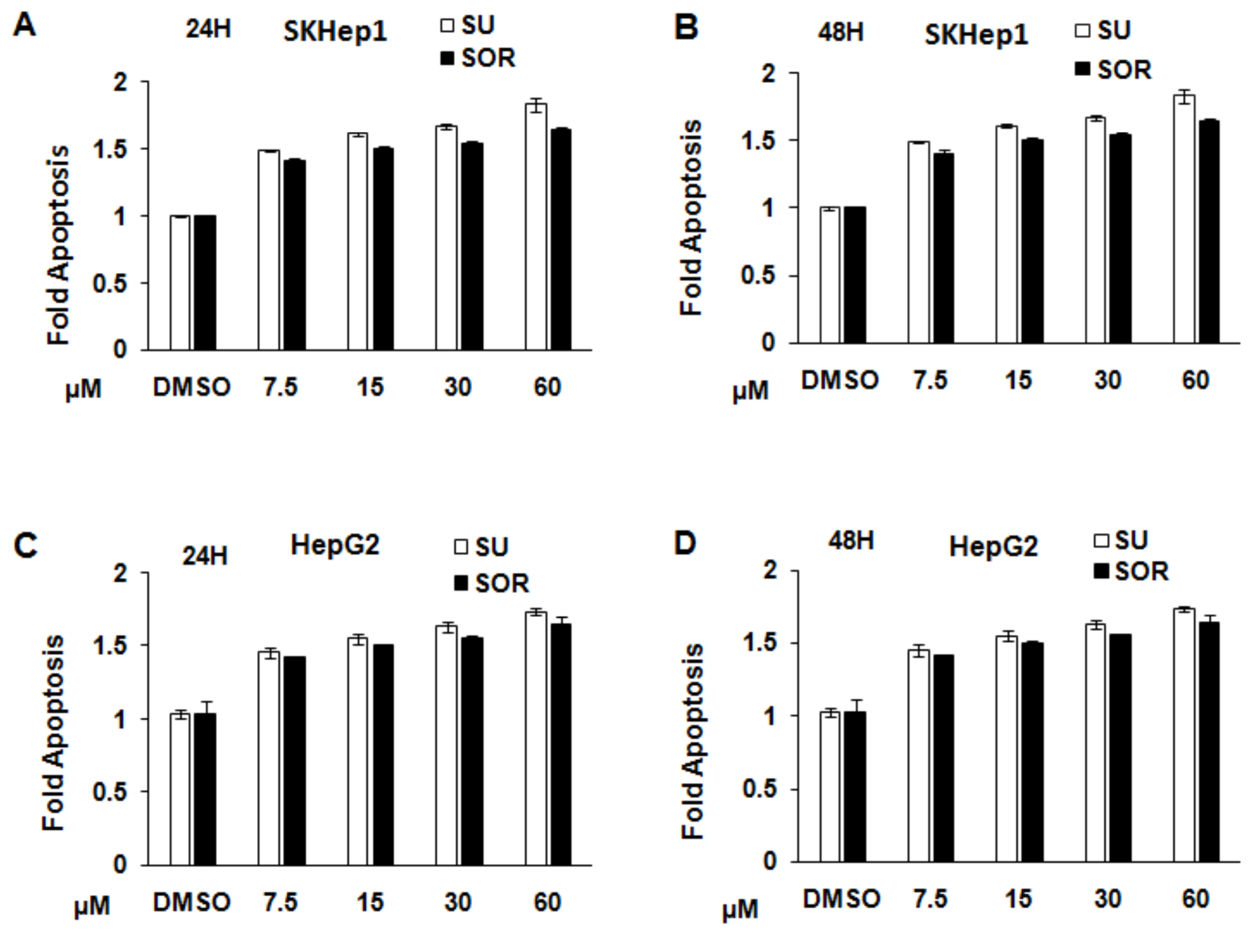
Sunitinib and sorafenib induce the apoptosis of human HepG2 and Sk-Hep-1 cells in a time- and dose-dependent manner HepG2, a human HCC cell, and Sk Hep 1, a human hepatic adenocarcinoma cell, were seeded into 96-well plates at 2 × 10^4^ cells per well in completed medium. The second day, sunitinib or sorafenib was added into each well at the indicated concentrations, DMSO as a vehicle solution was used as control. 24 or 48 hours after the treatment, cell apoptosis of Sk Hep1 (A, B) and HepG2 (C, D) were measured with Apo-one Homogeneous Caspase-3/7 Assay (Promega) according to the manufacturer’s instructions. The results are presented as a apoptosis fold of control cells treated with vehicle DMSO. n=3; error bars represent means ± S.D.

To compare the cytotoxic property of sunitinib and sorafenib in HCC and hepatoma cells, 2 × 10^4^ cells were seeded and cultured in each well of 96-well plate. 24 or 48 hours post treatment, proliferation and apoptosis of the cells with indicated treatments were measured. The results were presented as the percentage of cells undergoing proliferation in comparison with control without treatment. As shown in Figure 1A–1D, the results indicated that sunitinib and sorafenib both inhibited the proliferation of two types of cells in a dose- and time-dependent manner. More suppressive effect was observed in HepG2 cells compared to Sk-Hep1 cells. For example, 5 μM sunitinib or sorafenib treatment for 24 hours led to the reduction of Sk-Hep1 cells to about 70%, however approximately 60% and even less proliferation were detected in HepG2 cells. Compared to sunitinib, sorafenib exerted more cytotoxic effect on these two cell types. For example, treatment of Sk-Hep1 or HepG2 with 5 μM sunitinib or sorafenib for 48 hours, about 60% or 50% proliferated cells were detected (Figure 1B). In contrast, only less than 30% proliferated cells were detected in HepG2 and Sk-Hep1 cells treated with sorafenib (Figure 1D). Correspondingly, treatment with the two chemotherapeutic drugs induced cell apoptosis in Sk-Hep1 and HepG2 cells in the time- and dose-dependent ways (Figure 2A–2D). The extent of induced apoptosis with these two drugs was very similar, no much difference of cytotoxic effect was observed between Sk-Hep1 cells and HepG2 cells. Together, FDA-approved sunitinib and sorafenib similarly exert cytotoxic activity on HCC cells.

### Sunitinib and sorafenib treatment resulting in frequency alteration of immune cell subsets in tumor-bearing mice

**Figure 3:**
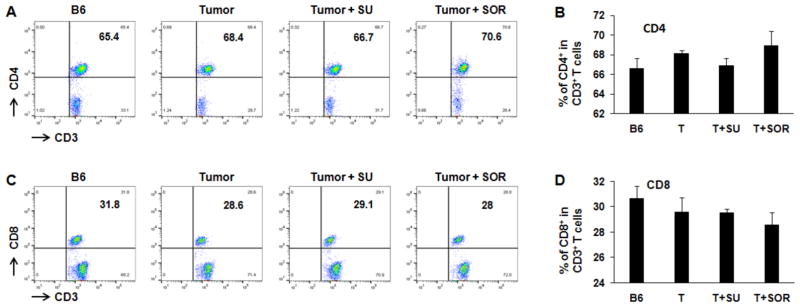
Treatment with sunitinib and sorafenib impacts the frequency of CD4^+^ and CD8 ^+^ T cells tumor-bearing mice Size-matched tumor-bearing mice received oral administration of sunitinib or sorafenib as described in methods. Lymphocytes were isolated from the spleens of each treated mouse, stained with fluorochrome-conjugated antibodies against CD3, CD4, and CD8, and then performed by flow cytometry. Splenic lymphocytes from normal mice and tumor-bearing mice without treatments were used for control. Representative and accumulated frequency of CD4^+^ T cells (A, B) and CD8^+^ T cells (C, D) are shown. n=3; error bars represent means ± S.D.

**Figure 4:**
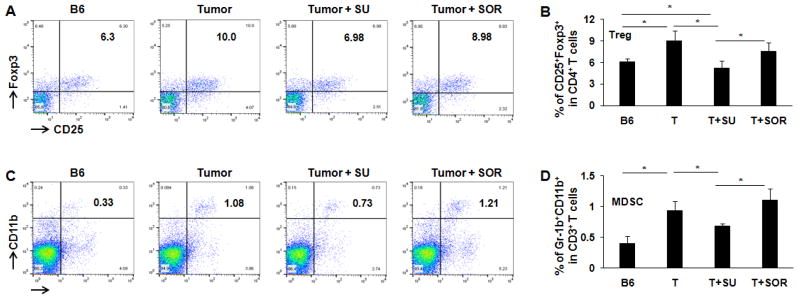
Treatment with sunitinib and sorafenib impacts the frequency of Treg and MDSC in tumor-bearing mice Size-matched tumor-bearing mice received oral administration of sunitinib or sorafenib as described in methods. Lymphocytes were isolated from the spleens of each treated mouse, stained with fluorochrome-conjugated antibodies against CD4, CD25, CD11b, Gr-1b, and CD11b, and then performed by flow cytometry. Splenic lymphocytes from normal mice and tumor-bearing mice without treatment were used for control. Representative and accumulated frequency of CD4^+^CD25^+^Foxp3^+^ Treg cells in CD4^+^ T cells (A, B) and Gr-1b^+^CD11b^+^cells in splenic lymphocytes (C, D) are shown. n=3; error bars represent means ± S.D.

In addition to the cytotoxic effect on tumor cells, we also explored and compared the role of sunitinib and sorafenib on immune system in tumor-bearing mice. First, we investigated whether sunitinib and sorafenib treatments differently modulate the frequencies of immune cell populations. Comparing control tumor-bearing mice without treatment, sunitinib administration led to the slight reduction in the frequency of CD4^+^ T cells from 68% to 67% (Figure 3A and 3B), but small increase of CD4^+^ T cells seen in sorafenib-treated mice from 68% to 70% (Figure 3C and 3D). No effect on frequency of CD8^+^ T cells was detected in either sunitinib- or sorafenib-treatment. Conversely, sunitinib treatment significantly reduced the magnitude of Tregs (CD4^+^CD25^+^FoxP3^+^) from 10% in control tumor-bearing mice without treatment to 7% (Figure 4A and 4B), and MDSCs (CD11b^+^Gr-1^+^) from 1.1% to 0.7% (Figure 4C and 4D). Sorafenib treatment led to the slight reduction of Treg frequency and small increase of MDSCs. Both changes were not statistically significant. These results suggest that sunitinib treatment reduces the frequency of immunosuppressive cell populations in the setting of HCC.

### Sunitinib and sorafenib treatment impact effector CD8^+^ T cell activity

**Figure 5:**
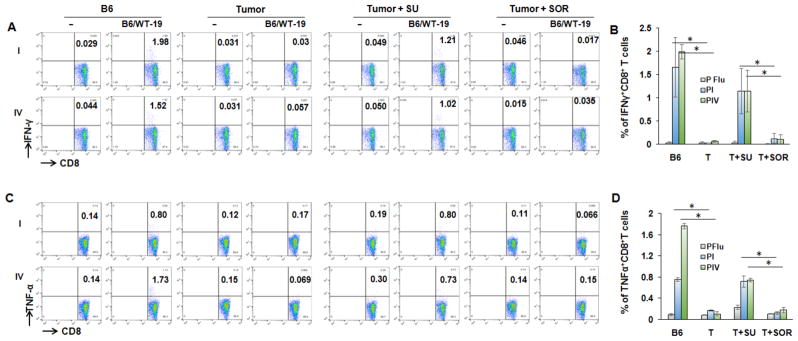
Sunitinib treatment activates CD8^+^ T cell activity in tumor-bearing mice The tumor-bearing mice were treated with sunitinib or sorafenib as described in methods, then immunized with 3 × 10^7^ of B6/WT-19 cells by intraperitoneal injection. Wild type C57BL/6 mice with or without immunization were used for control. On day 7 after immunization, the lymphocytes were isolated from the spleen of the immunized mice, and stimulated with 2 μM of peptide I, peptide IV or control peptide flu for 4 hours. After that, the cultured cells were stained with fluorochrome-conjugated antibodies against CD3, CD8, and IFN-γ and then conducted flow cytometry analysis. The representative (A) and accumulated (B) frequency of IFNγ^+^CD8^+^ T cells in the gated CD3^+^CD8^+^ T cells in the mice with the indicated treatment. The representative (C) and accumulated (D) frequency of TNFα^+^CD8^+^ T cells in the gated CD3^+^CD8^+^ T cells in the mice with the indicated treatment.. n=3, the error bar represent mean ± SD. Asterisks represent statistically significant differences (p<0.05).

To investigate if treatment with sunitinib or sorafenib is able to improve antitumor function, tumor-bearing mice were divided into three groups and receive vehicle, sunitinib, and sorafenib treatment, respectively. Following the indicated treatments, half of mice in each group received tumor antigen-specific immunization with transgenic B6/WT-19 cells expressing TAg. Wild type mice with or without immunization were used for control. Splenic lymphocytes were isolated from each mouse seven days post immunization and were stimulated with TAg epitope-I or –IV. The resultant production of IFN-γ and TNF-α in effector CD8^+^ T cells were measured with flow cytometry. As shown in Figure 5, epitope-I and epitope-IV were both unable to stimulate the production of IFN-γ and TNF-α in effector CD8^+^ T cells in vehicle- and sorafenib-treated tumor-bearing mice no matter whether they received immunization and not. Conversely, sunitinib treatment activated CD8^+^ T cells from immunized tumor-bearing mice, allowing epitope-I and -IV to effectively stimulate CD8^+^ T cells producing IFN-γ and TNF-α. The levels were equivalent to that seen in the immunized wild type mice. These results suggest that sunitinib treatment restores the activity of effector CD8^+^ T cells in HCC.

### Combination of sunitinib or sorafenib with TCR-I T cells plus immunization in the treatment of HCC

**Figure 6:**
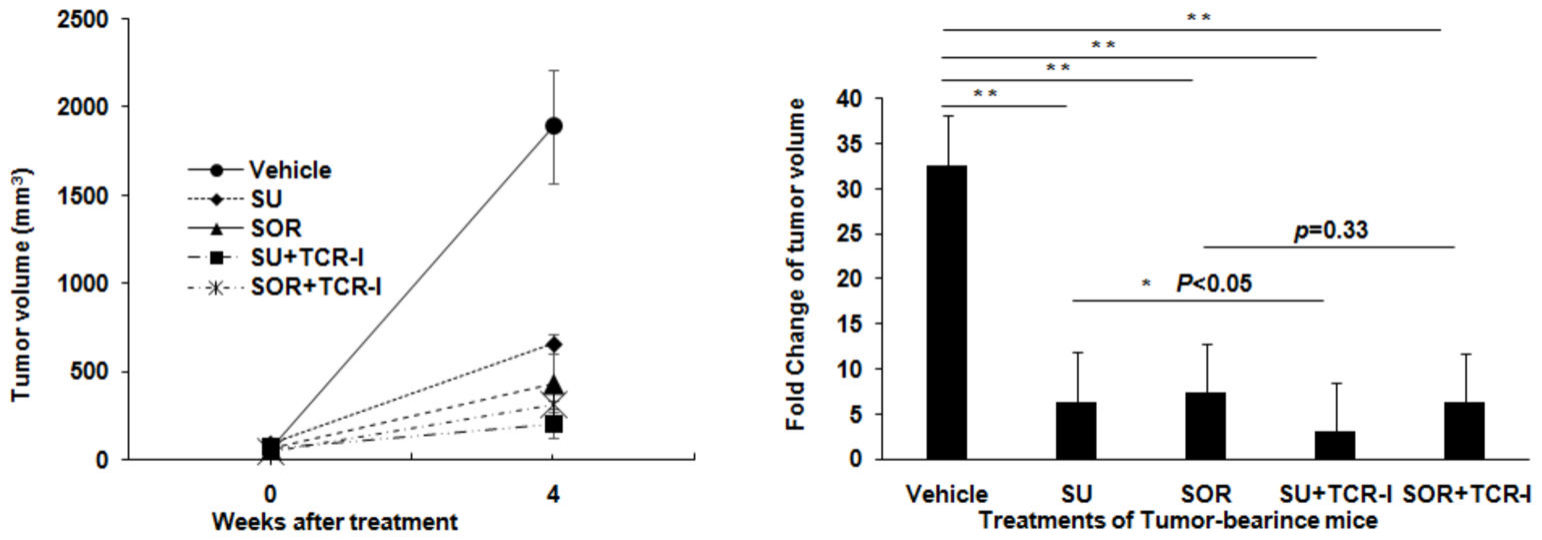
Combination of Chemotherapy and immunotherapy in HCC treatment Mice bearing size-matched established tumors were randomly assigned to five groups and received the indicated monotherapy and combined therapy. Tumor size in each mouse was measured with MRI 4 weeks after initial treatment with MRI. (A) Mean tumor volume over the time of the treatments. (B) Fold increases of tumors over the time of treatments. n = 5, the error bar represent mean ± SD. * *p*<0.05, ** *p*<0.01

To further investigate combination of chemotherapy and immunotherapy in the treatment of HCC, size-matched tumor-bearing mice were divided into five groups and received the following treatments: vehicle control, sunitinib monotherapy, sorafenib monotherapy, sunitinib or sorafenib combination with TCR-I T cells. All of mice were given the tumor antigen-specific immunization with transgenic B6/WT-19 cells expressing TAg. Four weeks after initial treatment, the tumor volume in each mouse was measured with MRI. We found that sunitinib and sorafenib monotherapies and their combination therapies effectively suppressed tumor growth. On week 4 after treatment, the mean tumor volumes were about 650 mm^3^, 430 mm^3^, 200 mm^3^, and 310 mm^3^ in each indicated group which were much less than 1800 mm^3^ seen in vehicle control mice (Figure 6A). The fold increase of tumor volume is 6.4, 7.4, 3.1, 6.2, respectively; all of them were much less than 32.7 fold seen in vehicle control group (Figure 6B). We observed addition of immunotherapy with TCR-I T cells and immunization to sunitinib monotherapy led to further suppression to tumor growth; but only minor effect was detected in sorfenib combination treatment. Together, combination of sunitinib and adoptive transfer of tumor antigen-specific CD8^+^ T cells is demonstrated to be an effective chemoimmunotherapic strategy, preventing HCC progression.

## DISCUSSION

In the present study, we compare the cytotoxic characteristic and immune modulatory effect between sunitinib and sorafenib in the context of HCC. Both of them maintain capability to inhibit growth of HCC cells and tumors. Interestingly, sunitinib shows a very strong immune modulatory effect in our clinically relevant murine model. As a result, combination of sunitinib treatment with external tumor antigen-specific CD8^+^ T cells plus immunization significantly suppress tumor progression (Figure 6). This effect is not detected in mice receiving sorafenib-integrated treatment. These synergistic results emphasize the combination of sunitinib with immunotherapy have a therapeutic potential in the treatment of HCC and sunitinib functions as an effective immune adjuvant to boost antitumor immune response.

Our findings demonstrate that’ sunitinib may be a preferable chemotherapeutic agent to use in combination with immunotherapy for the treatment of HCC [22]. While sunitinib and sorafenib have similar structure and tumoricidal effect, their effect on immune system is obviously different. We demonstrate that sunitinib treatment results in the significant reduction in the frequency of Treg and MDSC (Figure 4), allowing activation of endogenous effector CD8+ T cells in response to the immunization with tumor specific antigens. The results support previous findings in several clinical trials. Brossart’s group [23] found that sorafenib, but not sunitinib, inhibits function of DCs, impaired DC’s ability to migrate and stimulate T-cell responses. In contrast, sunitinib treatment reduced regulatory T cells in peripheral blood mononuclear cells. Van Herpen [24] enrolled 40 subjects in their clinical trial. 16 RCC patients were treated with sunitinib, 6 patients with sorafenib, 7 non treated controls, and 11 healthy control. Although all patients receiving sunitinib or sorafenib developed seroprotection to influenza vaccination comparable with controls, functional T-cell activity was only observed in three groups, rather than patients treated with sorafenib, evidenced by a decreased proliferation rate and IFN-γ/IL-2 production and increased IL-10 level compared with healthy controls. Salih’s group reported that pharmacological concentrations of sorafenib, but not sunitinib, inhibited cytotoxicity and cytokine production of resting and IL-2-activated PBMC (25), as sorafenib impaired reactivity of NK cells. NK cells substantially contribute to antitumor immunity by directly killing target cells and shaping adaptive immune responses through secreting cytokines like IFN-γ. These data suggest that sunitinib is able to activate immune response by modulating different immune cell subsets. In contrast, Perez-Gracia et al. reported that sorafenib was able to block VEGF-mediated impairment on DCs derived from normal persons through inhibiting its differentiation and maturation; however, this effect was not seen in sunitinib [26]. These data imply that sunitinib might only modulate DCs from patients.

Our studies also support findings from an open-label, phase III study which compares sunitinib versus sorafenib in advanced HCC [19]. The investigators reported that sorafenib may be safer and more effective than sunitinib as a monotherapy. We demonstrated that *in vivo* treatment of tumor-bearing mice with sunitinib and sorafenib monotherapy at same concentrations slowed down tumor growth with stronger effect seen in sorafenib (Figure 6). *In vitro* experiments suggested that this effect was mediated by suppressing tumor cell proliferation (Figure 1) and inducing tumor cell apoptosis (Figure 2). While the efficacy of inducing apoptosis with sunitinib and sorafenib was similar, more suppressive effect on HCC cell proliferation was detected in sorafenib.

In summary, sunitinib and sorafenib, as FDA-approved chemotherapeutic agents, differently impact antitumor immunity in the setting of HCC. Pretreatment of tumor bearing mice with sunitinib is able to prevent tumor-induced immunotolerance, activating tumor antigen-specific T cells to suppress tumor growth. Thus, integration of sunitinib and immunotherapy may be an effective therapeutic modality which can be translated into clinical practice of HCC. We will apply for a clinical trial to explore sunitinib-immunotherapy regimens in the treatment of patients with HCC and elucidate the underlying mechanisms.

## Abbreviations

CCl_4_: Carbon tetrachloride
CTLA-4: Cytotoxic T-lymphocyte-associated Protein 4
HCC: Hepatocellular Cancer
IP: Intraperitoneal
ISPL: Intra-splenic
MDSC: Myeloid-derived Suppressor Cell
MRI: Magnetic Resonance Imaging
PD-1: Programmed Cell Death Protein 1
PD-L1: Programmed Death-1 Ligand
RCC: Renal Cell Cancer
SOR: Sorafenib
SU: Sunitinib
TAg: SV40 T antigen
TCR: T Cell Receptor
